# Recurrent, ICD-associated *L. monocytogenes* bacteraemia with multiple septic pulmonary embolisms over a 2-year period

**DOI:** 10.1007/s15010-024-02209-w

**Published:** 2024-03-06

**Authors:** Astrid Füszl, Stefanie Schindler, Florian Heger, Mateusz Markowicz, Alexander Indra, Ariane Pietzka, Patrick Hyden, Adriana Cabal, René R. Wenzel

**Affiliations:** 1 National Reference Centre for Listeriosis, Austrian Agency for Health and Food Safety (AGES), Vienna, Austria; 2 Paracelsus Medical University Salzburg, Salzburg, Austria; 3 National Reference Laboratory for Listeria monocytogenes, Austrian Agency for Health and Food Safety (AGES), Graz, Austria; 4 Department of Statistics and Analytical Epidemiology, Austrian Agency for Health and Food Safety (AGES), Vienna, Austria; 5 Department of Internal Medicine (Cardiology & Nephrology), Tauernklinikum Zell am See, Salzburg, Austria

**Keywords:** Invasive listeriosis, Chronic listeriosis, *Listeria monocytogenes*, Implantable cardioverter-defibrillator, ICD, Relapse, Septic pulmonary embolism, cgMLST

## Abstract

**Background:**

*Listeria monocytogenes* is a bacterial pathogen known for causing listeriosis, a foodborne illness with a wide spectrum of clinical presentations ranging from mild gastroenteritis to severe invasive disease, particularly affecting immunocompromised individuals, pregnant women, newborns, and the elderly. Successful treatment of patients with recurring listeria episodes due to colonised foreign material is often challenging, typically requiring a combination of antimicrobial treatment and surgical removal.

**Case presentation:**

Here, we present a particularly complex case of chronic invasive listeriosis with a total of six relapses. After extensive investigations, the patient's ICD device was identified as the focus of infection.

**Conclusion:**

The confirmation of relapses through cgMLST analysis highlights the persistence of *Listeria monocytogenes* and the potential for recurrence even after apparent resolution of symptoms in patients with foreign material. It emphasises the necessity for a comprehensive assessment to identify and mitigate the risk of relapses, thereby ensuring optimal management and outcomes.

**Supplementary Information:**

The online version contains supplementary material available at 10.1007/s15010-024-02209-w.

## Introduction

*Listeria (L.) monocytogenes*, a gram-positive facultative anaerobic rod, is an opportunistic pathogen found ubiquitously in the environment. It is primarily transmitted through the consumption of contaminated food (e.g., unpasteurized dairy products, smoked salmon, poultry) [1, 2]. In neonatal infections, transmission occurs via the transplacental route, during childbirth when passing through the birth canal, or postnatally through contact. In immunocompetent individuals, the infection often remains asymptomatic or causes mild symptoms, for example, self-limiting gastroenteritis. In elderly or immunocompromised people, such as patients receiving immunosuppressive treatment, it can cause invasive listeriosis, most commonly manifesting as septicaemia or meningoencephalitis. Perinatal infection can result in abortion, stillbirth, generalised infection (granulomatosis infantiseptica) of the newborn, sepsis, or meningitis [1]. Less frequently, *L. monocytogenes* causes focal infections such as endocarditis, osteomyelitis, endophthalmitis or septic arthritis [3–5]. In rare cases, invasive listeriosis may involve multiple relapses, with native or prosthetic valves, intracardiac devices, or prosthetic joints serving as endogenous reservoirs [6–9].

This report details an unusual case of relapsing invasive listeriosis stemming from a colonised implantable cardioverter-defibrillator (ICD), which resulted in recurrent bacteraemic episodes and multiple pulmonary embolisms (PE) over a 2-year period.

## Case report

### Episodes I–IV in 2021

In March 2021, a 78-year-old female was admitted to the internal medicine department with fever, dyspnoea, hypotension, and an elevated heart rate. The patient’s medical history included an ICD device implantation in 2002 due to suspected Brugada syndrome with a lead replacement in 2011 and chronic diverticulitis. Sepsis caused by *L. monocytogenes* and bilateral PE were diagnosed. Enoxaparin treatment 2 × 1 mg/kg body weight s.c. was initiated, followed by dabigatran therapy 2 × 150 mg p.o. No precise information on the administered antimicrobial therapy could be retrieved.

In April 2021, she was readmitted with a Listeria-induced abscess in her right thigh, necessitating evacuation. *L. monocytogenes* was detected again in blood cultures. Simultaneously, there was another episode of PE with signs of right ventricular dysfunction. The patient’s ICD device or colonic diverticula were considered potential sources of infection. However, the transoesophageal echocardiography (TEE) showed no clear evidence of vegetations. Under dual antimicrobial therapy with piperacillin/tazobactam 3 × 1 g and gentamicin 160 mg i.v., follow-up blood cultures showed no further growth and inflammatory parameters normalised. Due to clinical improvement and unremarkable TEE findings, an extraction of the ICD device was not performed. The patient was discharged in early June in an improved condition. Subsequent outpatient PET/CT and colonoscopy appointments were arranged.

In late June 2021, during a routine check-up, *L. monocytogenes* was again isolated from blood cultures. The 18F-FDG PET/CT identified a suspicious region in a diverticula-bearing colonic segment, but no significant increase in FDG uptake along the ICD leads. To remove the suspected focus of infection, sigmoidectomy was performed. The histological analysis of the resected tissue revealed an inflammatory process. Microbiological cultures were not obtained. The patient received a 1-month treatment course with ampicillin 2 × 2 g and gentamicin 160 mg i.v. before being discharged in an improved general condition.

In August 2021, just 11 days later, the patient was readmitted with blood cultures yielding once again growth of *L. monocytogenes*, prompting a 10-day treatment with ampicillin 2 × 2 g and gentamicin 160 mg i.v., followed by the initiation of long-term suppressive treatment with amoxicillin 2 × 1000 mg p.o.

### Episode V in 2022

In September 2022, after a year of antimicrobial prophylaxis with consistently negative blood cultures, a withdrawal trial led to another bacteraemic episode accompanied by a PE. Treatment involved benzylpenicillin as well as a two-time administration of dalbavancin (1 × 1000 mg, 1 × 1500 mg) i.v., and a long-term prophylaxis with phenoxymethylpenicillin 3 × 1.5 g p.o. Anticoagulation with dabigatran 2 × 150 mg p.o. was replaced by apixaban 2 × 5 mg p.o.

By now, the ICD leads were believed to be the source of the recurrent infection, although the TEE findings remained unremarkable. However, surgeons were reluctant to extract them due to the risk associated with the intricate nature of the procedure and the potential complications involved.

### Episode VI in 2023

In February 2023, the patient presented with dyspnoea, weight gain, and peripheral oedema due to acute decompensated heart failure. Despite antimicrobial suppressive therapy, blood cultures once more yielded growth of *L. monocytogenes*. The antimicrobial regimen was modified to ampicillin 2 × 2 g and gentamicin 160 mg i.v., followed by ampicillin/sulbactam 2 × 220 mg/147 mg p.o. Due to the chronic nature of the infection, an interdisciplinary decision was eventually made to explant the ICD device.

In March 2023, PET/CT findings showed no pathological FDG hypermetabolism along the ICD leads or elsewhere. A transvenous extraction was attempted at a cardio-thoracic surgery centre, but the fragile ICD leads broke, preventing their complete removal. During the procedure, the patient developed cardiorespiratory instability due to extensive pulmonary emboli. A few days later, the patient experienced a stroke. Due to an unfavourable prognosis, it was decided to provide comfort terminal care, and the patient eventually passed away. Microbiological cultures from the explanted parts of the ICD yielded growth of *L. monocytogenes*.

Supplementary Fig. 1 provides a timeline summarising the patient’s bacteraemic episodes, diagnostic investigations, and treatment from March 2021 to March 2023.

## Microbiological investigations

All available *L. monocytogenes* isolates from this patient underwent additional microbiological investigations. These included antimicrobial susceptibility testing (AST), serotyping, and sequencing at the National Reference Centre for Listeriosis and the National Reference Laboratory for Listeria monocytogenes.

### Susceptibility testing

Susceptibility testing was performed on all isolates obtained from the patient and interpreted according to the recommendations of the European Committee on Antimicrobial Susceptibility Testing (EUCAST) [10]. ETEST® strips (bioMérieux, Marcy-l’Étoile, France) were used to determine the minimum inhibitory concentration (MIC) values of benzylpenicillin, ampicillin, meropenem, erythromycin and trimethoprim/sulfamethoxazole. All *L. monocytogenes* isolates were susceptible to the five antibiotics listed above (see Supplementary Table 1 for MIC values).

### Molecular analysis

To examine the phylogenetic relationship among isolates cultured during bacteraemic episodes from 2021 to 2023, high molecular weight DNA was extracted using the MagAttract HMW DNA Kit according to the manufacturer’s instructions (Qiagen, Hilden, Germany). Sequencing libraries were prepared using NexteraXT chemistry (Illumina Inc., San Diego, CA, USA) for a 2 × 300 bp sequencing run on an Illumina MiSeq sequencer.

Both Ilumina short-read sequencing and Oxford Nanopore Technology (ONT) long-read sequencing were performed, and the results were compared. ONT reads were filtered using filtlong 0.2.1 (parameters -t 270000000 -- min_length 1000 -- keep_percent 95). Hybrid assemblies were created using Flye (v. 2.9.2) (ref: 10.1038/s41587-019-0072-8) followed by polishing using medaka (v.1.8.0) and polypolish (v.0.5.0). Default parameters were used unless specified otherwise. Strains were typed using RidomSeqSphere + Software (Münster, Germany) core genome multilocus sequence typing (cgMLST) as described elsewhere [11]. The threshold used to define clonality in cluster analysis currently sets an allelic difference of seven as the cut-off [12].

**Fig. 1:**
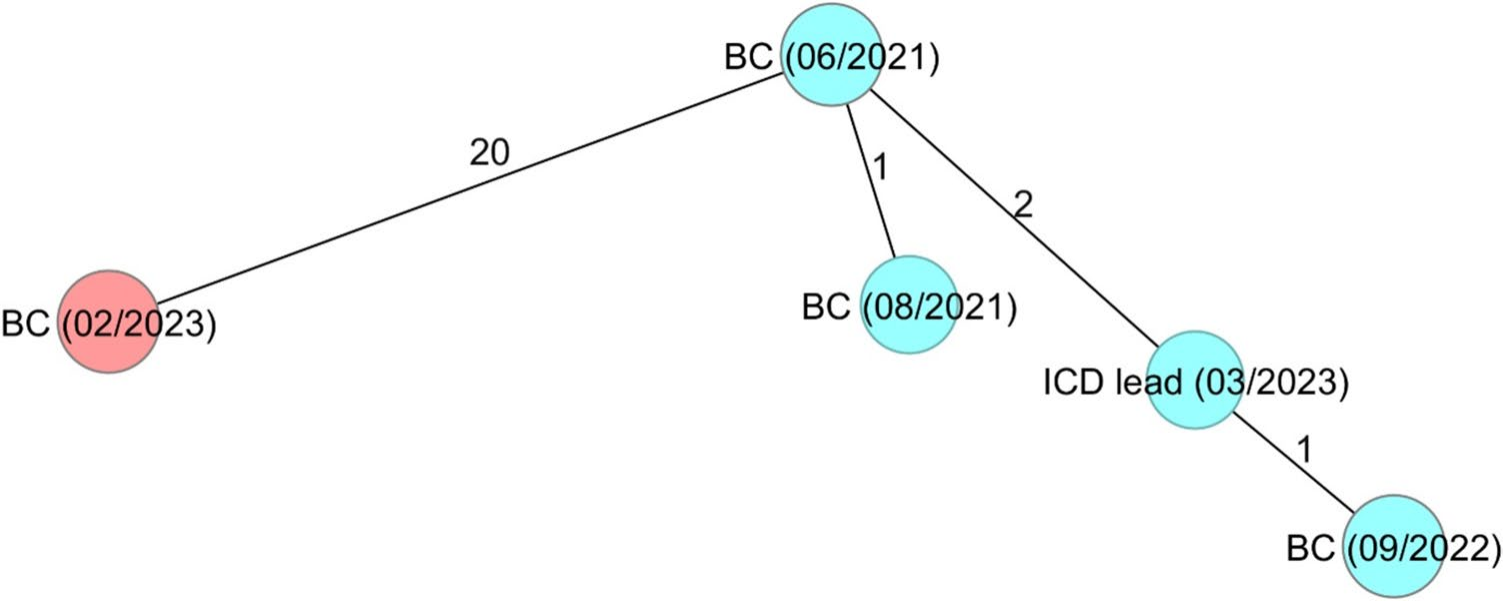
Minimum-spanning tree based on cgMLST allelic profiles of five *L. monocytogenes* isolates from the patient. Each circle represents a given allelic profile. The numbers on the connecting lines illustrate the number of differing alleles. Two different colours are used to distinguish the mutated patient isolate with > 20 alleles difference from the rest; BC = isolate from blood culture with the date of sample collection in brackets, ICD lead = isolate from implantable cardioverter-defibrillator lead with the date of sample collection in brackets

The results of the short- and long-read sequencing were identical, as summarized in Fig. 1. The three *L. monocytogenes* isolates obtained from blood cultures in 2021 and 2022, as well as the isolate obtained from the extracted ICD lead in 2023, exhibited a maximum of two allelic differences and were, therefore, considered a cluster (turquoise cluster). In contrast, the isolate from the most recent blood culture in 2023 differed from the other four isolates by 20 alleles (red colour).

Further analysis revealed that the BC isolate from 2023 showed several variations to the other isolates in the core genome and accessory genome, respectively, attributable to 22 probably defective genes in the isolate from 2023 (poly-A or poly-T indels, respectively) and to 41 genes in total (one SNP each). Notably, the 22 defective genes included lmo1232 (“recombination and DNA strand exchange inhibitor protein”) and lmo1403 (“DNA mismatch repair protein mutS”), both involved in DNA repair.

Additionally, the reconstructed genomes of the isolates from 2023 were shorter by 39–49 kb compared to the isolates from 2021 and 2022. Through pairwise BLAST alignment, it was observed that a 43.151 bp long fragment was missing in the isolates from 2023. This fragment was compared to the NCBI-nt database (viruses) and exhibited approximately 97% identity over 86% of the sequence to “Listeria phage LP-HM00113468, complete genome” (Accession NC_049900.1).

**Fig. 2:**
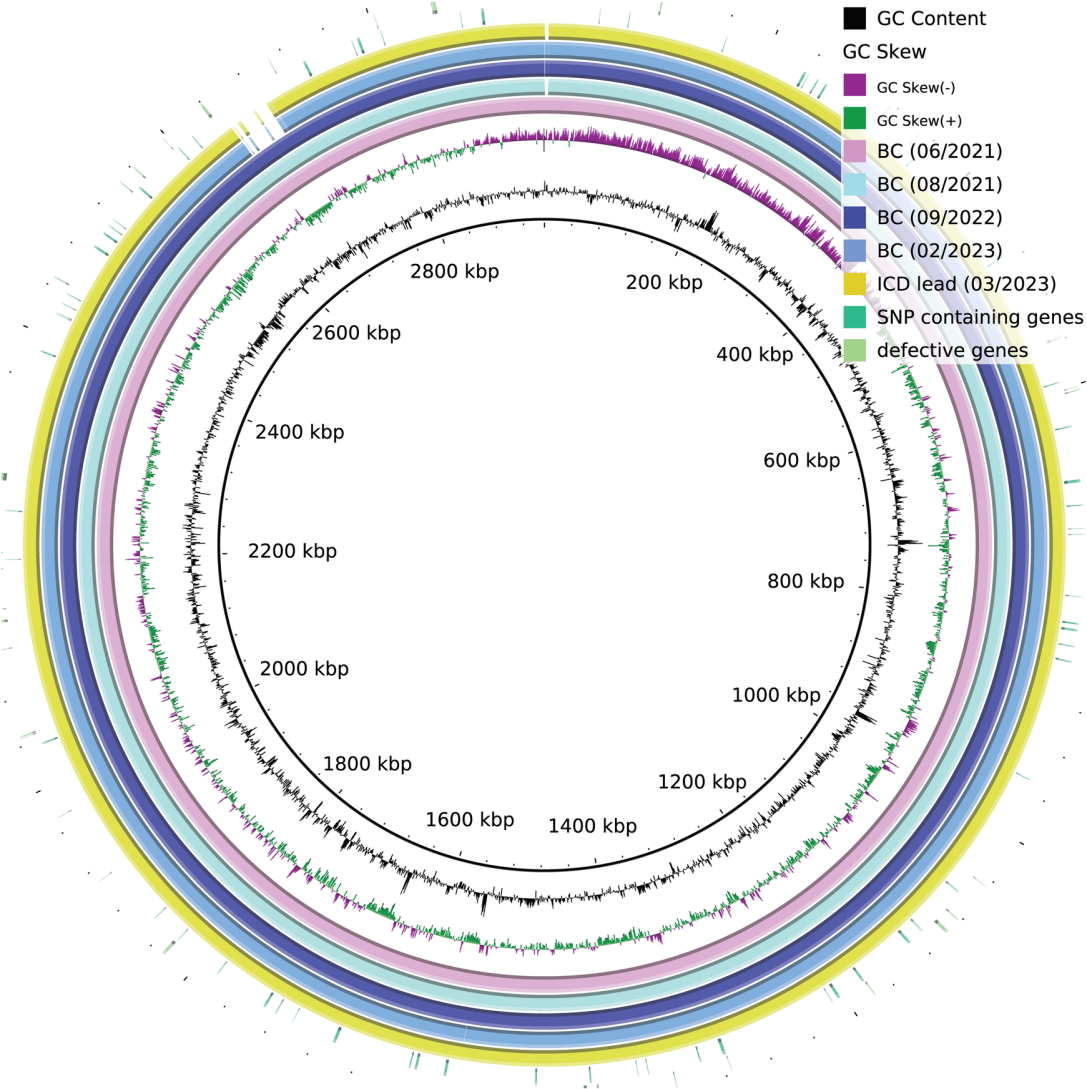
The reconstructed genomes from the 2023 isolates differ in a pro-phage, which is missing in the 2023 isolates (BC and ICD lead). Both SNPs (green) and defective genes (light green) are evenly distributed throughout the genome (two outer layers). The figure was created using Blast Ring Image Generator

Figure 2, generated with BRIG (v 0.95-dev.0004), shows that mutations (SNPs or defective genes through indels) are evenly distributed throughout the genome. The 43 kb long phage sequence integrated into the genome is depicted as a gap in the isolates from 2023.

Alignment of the patient’s sequences with the Listeria genome database of the National Reference Laboratory for Listeria monocytogenes (which currently contains > 18,000 sequences) did not identify any genetically closely related food or food-associated isolates.

## Discussion

This is a unique case of chronic *L. monocytogenes* infection likely originating from a colonised ICD device. The patient experienced six confirmed invasive listeriosis episodes, manifesting as positive blood cultures in 03/2021, 05/2021, 06/2021, 08/2021, 09/2022 and 02/2023. The initial source of the infection remains unclear, with a possible link to the consumption of salads containing raw flowers collected from a cow pasture, although no food samples were analysed.

To assess clonality, isolates underwent cgMLST typing. The isolates from the 2021 and 2022 blood cultures and the 2023 ICD lead showed identical profiles (allelic difference 1–2 alleles). However, the isolate from the most recent 2023 blood culture exhibited a distinct profile, differing in 20 cgMLST alleles. This was unexpected, as an allelic difference of 20 contradicts the definition of clonality, which sets a threshold of less than 7 cgMLST alleles [12]. Subsequent SNP analysis revealed that this discrepancy resulted from defects in two genes responsible for repairing mutations, having an accumulative effect. We hypothesise that this resulted in the formation of a distinct cluster, although the 2023 blood culture isolate remains part of the original clone. Therefore, the bacteraemic episode was likely caused by a relapse stemming from the colonised ICD leads rather than a reinfection.

While *L. monocytogenes*-associated endovascular infections (positive tissue samples with or without concomitant bacteraemia) originating from infected vascular aneurysms, prosthetic or native valves have previously been described [5], reports on intracardiac devices as sources are limited. Sharma and Yassin published one of these reports, presenting a case with a single Listeria-related bacteraemic episode linked to a pacemaker lead infection that was managed conservatively with vancomycin i.v. for 6 weeks followed by amoxicillin p.o. [13]. Other reports detail *L. monocytogenes*-associated relapses stemming from infected valves or aortic grafts [6, 9], with the case described by Rohde et al. experiencing two *L. monocytogenes* episodes within 7 weeks [9]. Clonal identity in this case was confirmed by pulsed field gel electrophoresis (PFGE). Surgical removal was impossible due to chronic aortic dissection. Conservative treatment included 6 × 2 g ampicillin i.v. for 6 weeks, complemented by gentamicin 240 mg daily for the first 4 weeks and oral amoxicillin 3 × 1 g for 8 weeks post-discharge, with no relapse over a 1-year follow-up. In another case, chronic *L. monocytogenes*-associated prosthetic joint infection occurred in a patient with knee and femur prostheses [7]. Knee aspirates repeatedly tested positive over a period of 18 months. Several courses of antimicrobial therapy and knee prosthesis replacement failed to achieve cure. Ultimately, resolution ensued following a 4-week regimen of linezolid, 3 months of rifampicin, and 4 months of co-trimoxazole. Although these cases demonstrate successful conservative approaches, numerous studies highlight that such strategies often lead to treatment failure, ultimately necessitating surgical graft, shunt or prosthesis removal [14]. In our patient, long-term antimicrobial prophylaxis based on susceptibility results also failed to prevent a relapse, eventually requiring a surgical intervention.

In the case reports mentioned above, imaging helped in the diagnostic workup; for instance, an echocardiogram showing signs of vegetation attached to the pacer lead [13], or a PET/CT showing an increased uptake in the aortic graft region [9]. However, in the case presented here, both TEE and PET/CT failed to identify the intracardiac device as the focus of infection. This discrepancy emphasises the importance of considering individualised diagnostic approaches based on the distinct characteristics of each case.

What is also noteworthy about this case is that it exhibited a recurrent pattern in which bacteraemic episodes coincided with pulmonary embolisms, potentially stemming from infected thrombi dislodging from the colonised ICD leads. More frequently, septic emboli associated with *L. monocytogenes*-related endocarditis have been described as the cause of strokes [5].

Interestingly, the patient lacked predisposing conditions often associated with invasive listeriosis, such as malignancy, immunosuppressive therapy, diabetes, cirrhosis, or kidney dysfunction [1]. However, as highlighted by Shoai-Tehrani et al. in their case series, factors beyond immunosuppression (e.g., the presence of prosthetic devices) may contribute to the occurrence of endovascular listeriosis [5]. Prosthetic devices carry the potential for biofilm formation [15], and infectious material from these biofilms may lead to hematogenous spread, causing multiple bacteraemic episodes as well as PE/strokes due to septic emboli. As pointed out by Shoai-Tehrani et al., individuals with *L. monocytogenes* bacteraemia who have such devices should undergo a comprehensive assessment for potential *L. monocytogenes*-associated prosthetic infection because they may require surgical intervention in addition to antimicrobial treatment.

In conclusion, we present a case where ICD leads acted as the endogenous reservoir for recurrent *L. monocytogenes*-related bacteraemic episodes accompanied by pulmonary embolisms. Antimicrobial treatment alone could not prevent relapses. However, surgical removal of the Listeria focus was associated with great risks, and the patient suffered a major PE during surgery and ultimately had a fatal outcome. Molecular typing was useful to understand the pathogenesis of this infection and the evolution of *L. monocytogenes* over time. A follow-up study is planned to elucidate the implications of our molecular findings for future *L. monocytogenes* cluster analysis, specifically addressing the current cluster threshold of seven alleles cgMLST difference.

### Supplementary Information

Below is the link to the electronic supplementary material.

Supplementary file 1 (PDF 209 KB)

## Data Availability

No datasets were generated or analysed during the current study.
