# Supplementary material for: Recurrent, ICD-associated L. monocytogenes bacteraemia with multiple septic pulmonary embolisms over a 2-year period
Source: Infection. 2024 Mar 6;52(4):1615–20. doi: 10.1007/s15010-024-02209-w (PMC11289073; doi:10.1007/s15010-024-02209-w)
Supplement: Supplementary file 1 — (PDF 209 KB) [file 15010_2024_2209_MOESM1_ESM.pdf]

Supplement

Recurrent, ICD-associated *L. monocytogenes* bacteraemia with multiple septic pulmonary embolisms over a 2-year period

Infection – A Journal of Infectious Diseases

Astrid Füszl[1,†,*], Stefanie Schindler[1,†], Florian Heger[1], Mateusz Markowicz[1], Alexander Indra[1,2], Ariane Pietzka[3], Patrick Hyden[4], Adriana Cabal[1], René R. Wenzel[5]

[1]National Reference Centre for Listeriosis, Austrian Agency for Health and Food Safety (AGES), Vienna, Austria
[2]Paracelsus Medical University Salzburg, Salzburg, Austria
[3]National Reference Laboratory for Listeriosis, Austrian Agency for Health and Food Safety (AGES), Graz, Austria
[4]Department of Statistics and Analytical Epidemiology, Austrian Agency for Health and Food Safety (AGES), Vienna, Austria
[5]Department of Internal Medicine (Cardiology & Nephrology), Tauernklinikum Zell am See, Salzburg, Austria

†These authors contributed equally to the paper.

***Corresponding author details:**

Phone: +43 664 88691875

E-mail: astrid.fueszl@ages.at

Supplementary Fig. 1

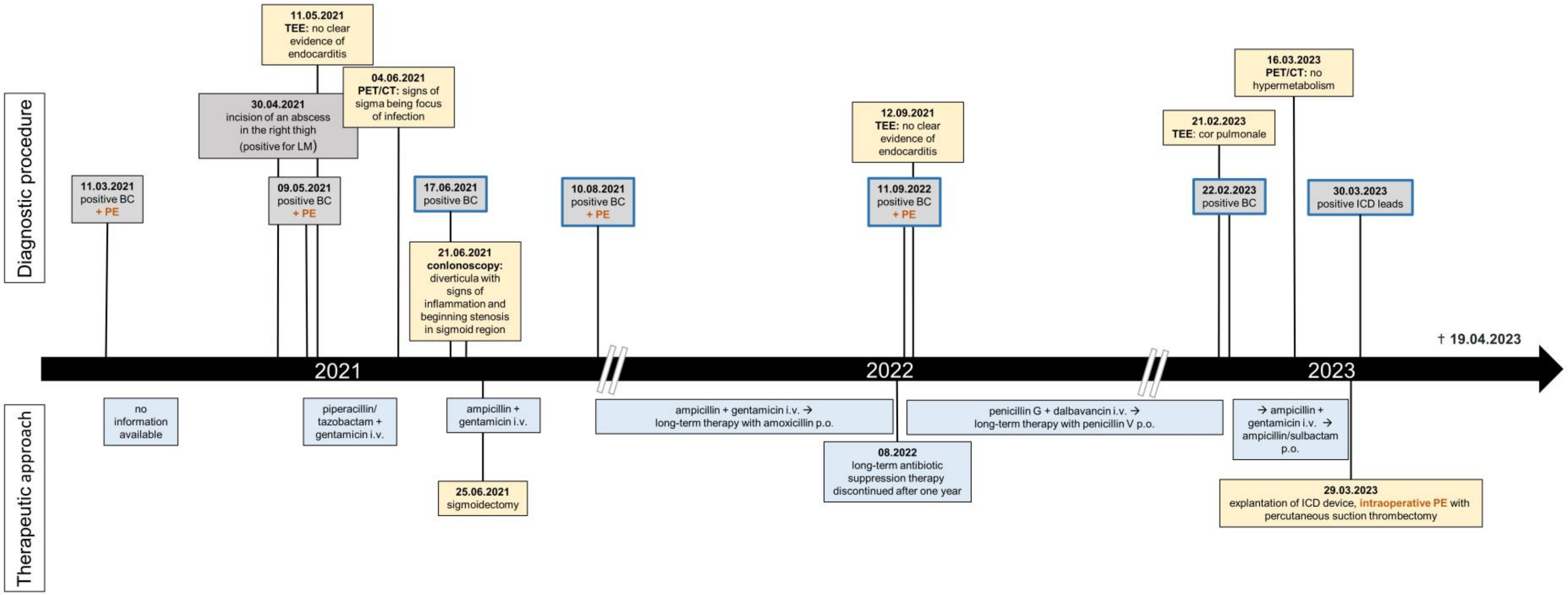

Timeline presenting the patient history (yellow boxes = imaging findings (top) and surgical procedures (bottom); blue boxes = antimicrobial therapy received; grey boxes = listeria detection; grey boxes with a blue frame = isolates available for microbiological and molecular evaluation; BC = blood culture; LM = *L. monocytogenes,* PE = pulmonary embolism, TEE = transesophageal echocardiography

Supplementary Table 1

Antibiotic agent	BC (06/2021)	BC (08/2021)	BC (09/2022)	BC (02/2023)	ICD lead (03/2023)
Benzylpenicillin	0.25	0.25	0.25	0.5	0.5
Ampicillin	0.50	0.38	0.19	0.75	0.75
Meropenem	0.094	0.19	0.094	0.125	0.125
Erythromycin	*	*	*	0.38	0.75
Trimethoprim/Sulfamethoxazole	*	*	*	0.023	0.032

Minimum inhibitory concentration (MIC) values in mg/L of five *L. monocytogenes* isolates obtained from the patient between 2021 and 2023; BC = isolate from blood culture with the date of sample collection in brackets, ICD lead = isolate from implantable cardioverter-defibrillator lead with the date of sample collection in brackets; *MIC values not available
